# Diabetes Mellitus and HIV Infection among Newly Diagnosed Pulmonary Tuberculosis Patients in the North West Region of Cameroon: A Cross-Sectional Study

**DOI:** 10.1155/2023/5998727

**Published:** 2023-11-24

**Authors:** Leonard Fonkeng Sama, Sidoine Sadjeu, Thibau Flaurant Tchouangueu, Solange Dabou, Georges Ful Kuh, Omer Bebe Ngouateu, Michel Noubom

**Affiliations:** ^1^Department of Biochemistry, University of Dschang, P.O. Box 67, Dschang, Cameroon; ^2^Gulf of Guinea University Institute, Institute of Applied Science, Douala, Cameroon; ^3^Department of Microbiology, Haematology and Immunology, Faculty of Health and Pharmaceutical Science, P.O. Box 96, University of Dschang, Dschang, Cameroon; ^4^University of Yaoundé I, Faculty of Science, Department of Animal Biology and Physiology, P.O. Box 812, Yaoundé, Cameroon

## Abstract

**Objective:**

To determine the prevalence rate of HIV and diabetes among tuberculosis (TB) patients and also the comorbidity rate.

**Design:**

Cross-sectional study. *Setting*. This study was carried out at the Tuberculosis Reference Laboratory, Regional Hospital Bamenda, North West Region of Cameroon, from January 2017 to December 2019. *Participants*. 1115 cases of pulmonary tuberculosis aged ≥14 years (mean 42.5 ± 15.28 years).

**Methods:**

Sputum samples collected were acid-fast stained and examined macroscopically as well as inoculated for culture. A chest X-ray was performed for further confirmation of TB diagnosis. After the TB diagnosis was done, fasting blood glucose, 2 h-PG test, HbA1c, and biochemical enzymatic tests were performed for the diagnosis of diabetes. Rapid strip test and enzyme-linked immunosorbent assay were used to diagnose HIV infection. *Interventions*. No intervention was done during the period of study. *Outcome Measures*. The prevalence of TB/HIV and TB/HIV/DM, signs and symptoms, imaging results, and bacteriology status among TB/HIV, TB/HIV/DM coinfected, and comorbidity cases.

**Results:**

Of 1115 participants, 38.57% had TB/HIV, and 5.83% had TB/HIV/DM. Among TB/HIV/DM cases, 20.39% had a cough for more than 2 weeks [*p*  <  0.0001; OR (95%CI): 4.866 (3.170–7.404)], and 35.71% had a fever for at least 2 weeks [*p*  <  0.0001; OR (95%CI): 7.824 (5.336–11.36)]. The majority of TB/HIV/DM patients (77.42%) had chest pain for at least 2 weeks [*p*  <  0.0001; OR (95%CI): 114.3 (59.78–207.1)]. 7.41%, 14.18%, and 9.09% of TB/HIV/DM, respectively, had chest abnormality, positive smear, and positive culture (*p* = 0.018). Significant differences were observed between signs and symptoms, imaging results, bacteriology, treatment history for TB cases and those with HIV and/or DM, and those without HIV and/or DM coinfection and comorbidity.

**Conclusion:**

This study reports a high prevalence of DM comorbidity and HIV coinfection among active TB patients in the North West Region of Cameroon as well as TB/HIV/DM comorbidity.

## 1. Introduction

Tuberculosis (TB) is an infection caused by the bacilli of the *Mycobacterium tuberculosis* complex which includes *Mycobacterium tuberculosis* stricto sensu, *M. bovis*, *M. africanum*, and *M. microti et M. pinnipedii*. It constitutes one of the ten leading causes of death in the world [1]. Despite the global strategy of treatment and prevention put in place by the World Health Organisation (WHO), the prevalence of TB is still rising [2]. The increase in the prevalence and speed of progression from latent tuberculosis (TB) infection to active TB is likely a consequence of immunodeficiency as a result of HIV infection [3] particularly in sub-Saharan Africa (SSA) where 95% of coinfection rates have been reported [4]. According to WHO, TB is one of the major causes of death among HIV-infected people, and TB/HIV coinfection has been found to reduce the effectiveness of directly observed therapy (DOT) of TB [5, 6]. The western regions of Cameroon particularly the North West Region has been identified as one of the regions where there is a rapidly increasing HIV epidemic that could fuel an epidemic of TB and also diabetes [7, 8]. Contrary to HIV and TB that are infectious diseases, diabetes is a metabolic disease characterised by chronic hyperglycaemia caused by lack or failure to use insulin or both [9]. These prevent glucose from getting into the cells for use as an energy source or to be stored. Diabetes is characterised by the metabolic disturbance which can result in a decrease in body defence against infection in general and in particular HIV and TB [10]. For this reason, the presence of TB and/or HIV in diabetic patients creates an environment for its propagation leading to serious complications. The incidence of diabetes varies from 2.1% to 38% according to studies and countries [11]. The increasing incidence of diabetes in the world constitutes by itself a great public health problem, and we can understand that its association with TB poses a big challenge to overcome. In view of this, there is a clear need to better understand the HIV and diabetes prevalence rate among TB patients and determine the coinfection and comorbidity rates in TB-infected individuals. The aim of this study was to determine the HIV and diabetes prevalence rates among TB patients as well as the coinfection and comorbidity rates.

## 2. Materials and Methods

### 2.1. Study Design and Location

This was a cross-sectional study conducted at the Regional Hospital Bamenda, North West Region of Cameroon.

### 2.2. Study Area and Period

The North West Region of Cameroon is situated in the high land of Cameroon and constitutes one of the 10 regions of Cameroon. The North West region has a surface area of 17300 km^2^, and it is divided into 7 divisions: Boyo, Bui, Donga-Mantum, Menchum, Mezam, Momo, and Ngo-Ketundja. The North West Region is situated at 1550m altitude above the sea level. The region has an average rainfall of 2567 mm, and temperature ranges from 19.3°C to 25.37°C. 88% of inhabitants practice farming and marketing of their products as the most important activities. Horticultural products include cabbage, carrot, onion, maize, banana, tomato, plantain, and beans. This study was conducted from January 2017 to December 2020. The Regional Hospital Bamenda is the reference and biggest hospital of the region with one TB unit and the second of the TB reference laboratories in Cameroon after “Centre Pasteur du Cameroun” in Yaounde. This TB reference laboratory covers other subunits of the West, North West, and part of the South West Regions of Cameroon.

### 2.3. Participant Selection and Criteria

All newly diagnosed positive cases for pulmonary TB were eligible for the study. Eligible patients who provided consent were enrolled in this study. A questionnaire on demographic and clinical data was administered to each participant.

All patients who had been on TB treatment or those experiencing multidrug resistance (MDR) were excluded because according to literature, TB treatment can increase impaired glucose tolerance that is common, and it is one of the factors that diabetes drugs are designed to control [12, 13]. A total of 1221 suspected patients were recruited in the study irrespective of age.

### 2.4. Sample Collection

After a brief training on how to collect the sample, sterile plastic cups (03) were given to each patient for sputum collection. The first sputum was collected immediately in the hospital after a brief training and the second early in the morning of the next day. 5 ml of blood was collected from positive cases into grey tubes and allowed to stand for few minutes. Thereafter, it was centrifuged at 3500 rpm for 15 min at 4°C. Then, the supernatant was collected and kept at −20°C for further testing.

### 2.5. Diagnosis of Tuberculosis

The diagnosis of tuberculosis was based on either a positive sputum smear, X-ray, and/or a positive culture using Löwenstein–Jensen media (Sino Biological Inc., India) that was prepared according to the manufacturer's instructions and poured inside the screw tube. The culture was considered positive when there were grown colonies whereas a negative culture was characterised by the absence of colony growth as shown by Somoskovi et al. [14].

### 2.6. Diagnosis of Type 2 Diabetes

The diagnosis of type 2 diabetes was done based on American Diabetes Association (ADA) guidelines of fasting blood sugar ≥126 mg/dl, 2 h plasma glucose (PG) ≥200 mg/dl, and glycated haemoglobin (HbA1c) assay with cut-off point 6.5% [9, 15]. This was further confirmed by the enzymatic method using a glucose oxidase/peroxidase kit (Sino Biological Inc., India).

### 2.7. HIV Testing

HIV testing was first performed using a strip test (Alere Determine™ HIV-1/2), and HIV-positive cases were confirmed by enzyme-linked immunosorbent assays (ELISA) (Sino Biological Inc., India).

### 2.8. Patient and Public Involvement

Research questions were generated based on general observation of patients suspected to suffer from TB. Priority was given to patients presenting symptoms that were characteristics of TB patients and were recruited to provide samples firstly to test for TB (sputum), then HIV (blood), and diabetes (blood). Tests were performed by the principal investigator, and their results were coded. When necessary, the result was returned to the consulting physician who did the prescription. Patients were not randomized.

### 2.9. Statistical Analysis

Data analysis was performed using GraphPad Prism software version 8.0.2. The prevalence of HIV and diabetes among the TB cases was estimated as a proportion with 95% confidence limits. The difference in outcomes for binary variables was established using the Pearson Chi-square test while the Kruskall–Wallis difference in proportions was used for multiple categorical variables. Results were considered statistically significant at *p* value <0.05.

## 3. Results

Out of 1221 TB-suspected patients who were enrolled in this study, 1115 were confirmed positive for TB. Their mean age is 42.5 ± 15.28 years, with an age range from 14 to 89 years. Males were significantly (*p*  <  0.0001) more represented compared to females (64.57% vs 35.43%). A significant difference (*p*  <  0.0001) was observed between the patients' age groups with those in the age group 31–40 being more in number. According to their educational level, patients with a primary level of education were significantly (*p*  <  0.0001) higher compared to those with secondary and higher levels of education. Considering the marital status, a significant difference (*p* = 0.0002) was observed between the overwhelming number of married patients compared to unmarried. Patients engaged in a physical activity lifestyle showed a significant difference (*p* = 0.0001) with patients physically nonactive being greater in number. Patients with no family history of diabetes were significantly (*p*  < 0.0001) more compared to those having a family history of diabetes.  Table 1.

Sociodemographic characteristics also showed that smokers were significantly (*p*  <  0.0001) more represented than nonsmokers whereas patients not consuming alcohol were significantly (*p*  <  0.0001) higher relative to alcohol consumers. Patients with normal Body Mass Index (BMI) were significantly more (*p*  <  0.0001) compared to underweight, overweight, and obese cases. A significant difference (*p*  <  0.0001) was noted between nondiabetic cases compared to diabetic patients. A significant difference (*p*  <  0.0001) was also observed between HIV-negative cases and HIV-positive cases as shown in Table 1.

The prevalence of TB/DM was found to be 12.11%, whereas the prevalence of TB/HIV and TB/HIV/DM was found to be 38.57% and 5.85% successively.

Among the participants who had a cough for more than 2 weeks, 20.39% were TB/HIV/DM [*p*  <  0.0001; OR (95%CI): 4.866 (3.170–7.404)]. For those with fever for at least 2 weeks, 35.71% were TB/HIV/DM [*p*  <  0.0001; OR (95%CI): 7.824 (5.336–11.36)]. A majority of TB/HIV/DM cases (77.42%) had chest pain for at least 2 weeks [*p*  <  0.0001; OR (95%CI): 114.3 (59.78–207.1)]. A magnitude of 7.41%, 14.18%, and 9.09% of TB/HIV/DM patients had chest abnormalities, positive smears, and positive cultures, respectively (*p* = 0.018), as presented in Table 2.

Table 3 highlights the signs, symptoms, imaging results, and bacteriology status for TB cases with and without HIV coinfection as well as those with and without diabetes. A significant difference (*p* <  0.0001) was observed among these groups: TB+/HIV+, TB+/DM+, TB+/HIV+/DM+, TB+/HIV−, TB+/DM−, and TB+/HIV−/DM− as well as TB+/DM− (79.61%) having cough ≥2 weeks. A significant difference (*p* <  0.0001) was also noted among TB+/HIV+, TB+/DM+, TB+/HIV+/DM+, TB+/HIV−, TB+/DM, and TB+/HIV−/DM− as well as TB+/DM− (64.29%) with fever ≥2 weeks. Among patients suffering from chest pain ≥2 weeks, a significant difference (*p* < 0.0001) was conspicuous among TB+/HIV+, TB+/DM+, TB+/HIV+/DM+, TB+/HIV, TB+/DM, and TB+/HIV−/DM− with 80% of TB+/DM− presenting these symptoms.

We also noticed in our study a significant difference (*p*  <  0.0001) in the diagnostic methods with 92.59%, 85.82%, and 90.91% of TB+/DM- manifesting, respectively, with chest X-ray abnormality, positive smear, and positive culture. A significant difference (*p*  <  0.0001) was also observed between patients with “household member ever treated for TB” and those with non-TB treated with 90.91% of TB+/DM− with “household member ever treated for TB”.

## 4. Discussion

The prevalence of HIV among the newly diagnosed TB cases tested in this study was 38.57%. This result was almost eight times higher than the 5.1% routinely reported among the national population [16]. In general, surveys in Africa, Asia, and the Pacific have indicated that the HIV prevalence among TB patients is much higher than that observed in the general population [17]. The prevalence found in our study is higher than that found in a systematic review and meta-analysis in sub-Saharan Africa where the overall pooled prevalence was 31.8%, and that observed in the Eastern and Southern sub-Saharan Africa region where HIV prevalence was 34.4% and 27.3% in Western and Central sub-Saharan Africa region [18]. This high prevalence could be due to the fact that the HIV epidemic has influenced significantly the epidemiology of TB. In addition, HIV is a well-known risk factor for progression to active TB among those infected with *Mycobacterium tuberculosis* [19]. This high prevalence of HIV could also be due to the fact that this study was carried out in the North West Region with the second highest prevalence of HIV infection after the South Region of Cameroon [20]. Furthermore, it is possible that TB/HIV coinfected individuals who fall sick are more likely to go to the health facility earlier because of the severity of symptoms and are thus potentially diagnosed earlier. The prevalence observed in our study was lower than that reported in a study conducted in the North West Region of Cameroon with an HIV prevalence of 68.5% among TB [21]. This difference could be due to the difference in the sample size that was almost more than twice our sample size. Additionally, their study sampled one public hospital (Regional Hospital Bamenda) and 3 faith-based hospitals unlike our study that focused only on the Regional Hospital Bamenda. This disparity can also be explained by the fact that the prevalence of HIV has reduced with time since a good progress has been made in implementing TB/HIV collaborative activities in Cameroon and the North West Region in particular. Unfortunately, in this survey, most of the HIV patients (20.51%) knew their status and were on anti-retro-viral (ARV) treatment. Since TB patients were newly diagnosed, all the TB individuals were later placed on TB treatment. However, newly diagnosed TB/HIV coinfected patients (18.06%) were placed on both TB and ARV treatment at the same time.

The prevalence of type 2 diabetes among the newly diagnosed TB cases in this study was 12.11%. This is the first large-scale study to examine the association of TB and type 2 diabetes in Cameroon. The prevalence obtained in this study is higher than the prevalence obtained in the first study conducted in Cameroon with a prevalence of 9.4% [22]. Though the study was conducted in the same area, the prevalence differences in these two studies could be due to differences in sample size which is larger in this study. It can also be due to the increase in diabetes in the general population [23]. According to a systematic review and meta-analysis conducted on the prevalence of diabetes mellitus (DM) among tuberculosis patients in sub-Saharan Africa based on a total of 63 full-text articles, the pooled prevalence of DM among TB patients was found to be 9% [24]. This difference with our prevalence data could be justified by the fact that the prevalence of DM varies from one country to another and from one region of the same country to another. The prevalence obtained from this study is in line with the estimated prevalence of DM (8.5–16.4%) among TB patients in sub-Saharan Africa [22, 25, 26].

The prevalence of TB/HIV/DM was found to be 5.83%. According to our knowledge, this is one of the few studies to examine the TB/HIV/DM association in Africa, one of the high-burden TB regions in the world. This result was slightly higher than the 4.8% reported in a study among active tuberculosis patients in Northwest Ethiopia [27]. This slightly high prevalence noticed in our study compared to that of Tulu et al. could be due to our study population which is almost five times larger than that conducted in Northwest Ethiopia [25]. In the systematic review and meta-analyses conducted by Al-Rifia et al. [11], it was proven that patients with DM had 3.59 times the risk of developing TB though the mechanism is not clear. HIV is a well-known risk factor for progression to active TB by compromising the immune system. The burden of DM and HIV among TB cases could be due to the fact that HIV compromises the immune system and leads to the progression to active TB among those infected with latent *Mycobacterium tuberculosis* [17]. The initiation of ARV treatment leads to rapid weight gain which is in correlation with an increase in insulin resistance, a cause of diabetes, [28] which in turn constitutes an increased risk of developing TB. According to Tutu et al., HIV/TB coinfection is a predictor of DM comorbidity in active TB patients [27].

Almost 8% (7.48%) of the patients having chest anomalies were TB/HIV/DM while 14.18% had positive smears and 9.09% had positive cultures. This may imply that chest X-ray screening of HIV/DM-infected and comorbid people for TB was possible though the prevalence of coinfected and comorbid cases with chest abnormalities was lower compared to the 88.9% observed in a study conducted in Zambia [4]. This difference could be due to the fact that patients in this study were in their early stages of TB.

The prevalence of patients with tuberculosis varies according to coinfection or comorbidity or not, with TB/HIV+ recording a higher prevalence of 59.52%. This possibly could be due to the fact that this study was conducted in an endemic area of malaria. The high prevalence observed among TB/HIV+ may also be explained by an HIV-compromised immune system and results in the progression of malaria among those infected with latent *Mycobacterium tuberculosis* [17]. Also, cases of coinfection of TB with intracellular parasites were reported to have malaria [29].

TB programs should continue to routinely screen for HIV and DM in order to improve TB/HIV/DM detection and management [30, 31]. On the other hand, HIV treatment centres and diabetes management centres should in fact scale up coscreening and comanagement of TB, TB/HIV, and TB/HIV/DM so that coinfected individuals and comorbidity can be provided with comprehensive care and management [29]. Integration of TB/HIV/DM diagnoses and treatment services in a one-stop-shop approach is key regardless of whether a patient's entry into the health system is through the TB or HIV or DM clinic because they have mutual goals to find and treat cases [32, 33]. However, rigorous evaluation of the impact of the various models of integration is required [33]. Cameroon should consider testing all presumptive TB cases for HIV and diabetes in a bid to implement a more sensitive case detection algorithm.

This is the first study documenting the prevalence of HIV/diabetes in the TB population in Cameroon.

The limitations of this analysis are that our study was focused only on the Regional Hospital Bamenda. Though it is a reference hospital of the region, it is not the only hospital where TB patients are managed and followed up. All patients suspected of/or having extra-pulmonary tuberculosis or MDR tuberculosis were excluded from this study, and this might have underestimated the true burden of TB/HIV/DM. The level of HIV on immune suppression was not also evaluated.

## 5. Conclusion

In conclusion, this study reports a high prevalence of DM comorbidity and HIV coinfection among active TB patients in the North West Region of Cameroon as well as TB/HIV/DM comorbidity. Thus, a collaborative approach should be implemented to improve the screening and management of DM and HIV among TB patients at the TB units.

## Figures and Tables

**Table 1:** Sociodemographic characteristics of the TB cases.

Variable	*N*	% (95% CI)	*p* value (Chi-2)
Sex			
Male	720	64.57	<0.0001^*∗*^
Female	395	35.43	
Age group			
<21	70	6.28	<0.0001 (550.7)
21–30	270	24.22	
31–40	365	32.74	
41–50	185	16.59	
51–60	115	10.31	
61–70	75	6.73	
>71	35	3.14	
Educational level			
Primary	615	55.16	<0.0001 (465.1)
Secondary	455	40.81	
Higher	45	4.04	
Marital status			
Married	620	55.61	0.0002^*∗*^
Unmarried	495	44.39	
Settled way of life			
Yes	645	57.85	<0.0001^*∗*^
No	470	42.15	
Smoking status			
Yes	820	73.54	<0.0001^*∗*^
No	295	26.46	
Alcohol intake			
Yes	390	34.98	<0.0001^*∗*^
No	725	65.02	
BMI			
Underweight	219	19.64	<0.0001 (1460)
Normal	816	73.18	
Overweight	60	5.38	
Obese	20	1.79	
Waist circumference			
>94 cm (male)	5	0.45	<0.0001 (1004)
<94 cm	715	64.13	
>80 cm (female)	170	15.25	
<80 cm	225	20.18	
Family history of diabetes			
Yes	355	31.84	<0.0001^*∗*^
No	760	68.16	
Diabetes mellitus			
Yes	135	12.11	<0.0001^*∗*^
No	980	87.89	
HIV serology status			
Positive	430	38.57	<0.0001^*∗*^
Negative	685	61.43	

^*∗*^Fisher's exact test was used to determine the difference between two variables at 95% CI, and Chi-squared values were not generated.

**Table 2:** Signs and symptoms, imaging results, and bacteriology among TB/HIV/DM coinfected and comorbidity cases.

Characteristics	Total	TB cases coinfected with HIV and comorbidity with DM	%	*p* value	OR (95% CI)
Cough ≥2 weeks					
Yes	515	105	20.39	<0.0001	4.866 (3.170–7.404)
No	600	30	5.00		
Fever ≥2 weeks					
Yes	210	75	35.71	<0.0001	7.824 (5.336–11.36)
No	905	60	6.63		
Chest pain ≥2 weeks					
Yes	155	120	77.42	<0.0001	114.3 (59.78–207.1)
No	515	15	2.91		
Diagnostic methods					
Chest X-ray abnormality	135	10	7.41	0.018	N/A
Positive smear	705	100	14.18		
Positive culture	275	25	9.09		
Household member ever treated for TB					
Yes	275	25	9.09	0.088	0.6636 (0.4150–1.053)
No	840	110	13.10		

**Table 3:** Signs and symptoms, imaging results, bacteriology, and treatment history for TB cases with HIV and/or DM and without HIV and/or DM coinfection and comorbidity.

	TB+/HIV+	N (%)	TB+/DM+	N (%)	TB+/HIV+/DM+	N (%)	TB+/HIV−	N (%)	TB+/DM−	N (%)	TB+/HIV−/DM−	N (%)	Total	*p*-value (Chi-square)
Number of cases	430	38.57	135	12.11	65	5.83	685	61.43	980	87.89	615	55.16	1115	
Cough ≥2 weeks (yes)	235	45.63	105	20.39	50	9.71	280	54.37	410	79.61	225	43.69	515	<0.0001 (369.9)
Fever ≥2 weeks (yes)	125	59.52	75	35.71	35	16.67	80	38.10	135	64.29	45	21.43	210	<0.0001 (310.8)
Chest pain ≥2 weeks (yes)	260	43.33	120	20.00	61	10.17	340	56.67	480	80.00	280	46.67	600	<0.0001 (148.0)
Diagnostic methods														
Chest X-ray abnormality	40	29.63	100	74.07	0	0.00	95	70.37	125	92.59	85	62.96	135	<0.0001 (398.1)
Positive smear	285	40.43	25	3.55	55	7.80	420	59.57	605	85.82	375	53.19	705	
Positive culture	105	38.18	10	3.64	10	3.64	170	61.82	250	90.91	155	56.36	275	
Household member ever treated for TB (yes)	60	21.82	15	5.45	61	22.18	215	78.18	250	90.91	200	72.73	275	<0.0001 (212.3)

## Data Availability

The datasets used and/or analysed during the current study are available from the corresponding author upon reasonable request.

## Additional Points

*What Is Known?* (i) In Cameroon, there is no universal screening for diabetes among the tuberculosis population and the potential manifesting factors associated with TB infection. *What Is New?* (i) This study builds on previous literature to differentiate signs and symptoms, imaging results, and bacteriology among coinfected and comorbidity cases. (ii) We found that cough ≥2 weeks, fever ≥2 weeks, chest pain ≥2 weeks, chest X-ray abnormality, positive smear, positive culture, and household member ever treated for TB were associated with TB patients coinfected and with comorbidity among TB, HIV, and diabetes.
